# Different in so many ways: Exploring consumer, health service staff, and academic partnerships in a research advisory group through rapid ethnography

**DOI:** 10.1111/1440-1630.12830

**Published:** 2022-07-24

**Authors:** Ruth Cox, Matthew Molineux, Melissa Kendall, Elizabeth Miller, Bernadette Tanner

**Affiliations:** ^1^ Occupational Therapy Department Queen Elizabeth II Jubilee Hospital Coopers Plains Queensland Australia; ^2^ Discipline of Occupational Therapy, School of Health Sciences and Social Work Griffith University Queensland Australia; ^3^ Acquired Brain Injury Outreach Service and Transitional Rehabilitation Program Princess Alexandra Hospital Buranda Queensland Australia; ^4^ School of Health Sciences and Social Work Griffith University Meadowbrook Queensland Australia

**Keywords:** consumer and community engagement, consumer participation, partnering with consumers, research advisory group

## Abstract

**Introduction:**

Consumer and community involvement (CCI) encompasses the range of consumer engagement activities across the research cycle. Research advisory groups (RAGs) are a common method of CCI that may empower the consumer voice in research. However, there is limited evaluation of RAGs to guide occupational therapists considering this as a CCI strategy in research. The aim of this study was to explore the processes and outcomes of a RAG partnership for an eDelphi study.

**Methods:**

Rapid ethnography enabled a rich, thick description of the RAG through triangulation of field notes, a monthly research team log, focus groups, and an individual interview. Data were analysed using reflexive thematic analysis. Recruitment targeted consumers, health service staff, and academics with experience in CCI to enhance the diversity of perspectives guiding the eDelphi study. The RAG met four times over 4 months.

**Findings:**

Seven diverse RAG members were recruited resulting in a RAG of 12 members, including the research team that included two consumers. Reflexive thematic analysis resulted in an overarching theme: *Different in so many ways*, which reinforced that authentic CCI in research continues to be rare even for stakeholders with experience in CCI. There were four subthemes: *Set up for success*, *Authentic and capable facilitation*, *Structures and strategies for genuine partnerships*, and *A ripple effect of benefits*. Findings added to the limited research regarding RAGs and highlighted that a short‐term RAG with 12 diverse stakeholders was an effective strategy to foster mutually beneficial and meaningful collaboration. Partnering with two consumer co‐researchers in RAG planning, implementation, and evaluation was central to success.

**Conclusion:**

Findings demonstrated that with careful co‐planning and recruitment, capable facilitation with support of a committed research team (inclusive of consumers), and empowering meeting processes and structures, a short‐term RAG resulted in many benefits to participants and enhanced research outcomes.

**Key Points for Occupational Therapy**

- Engagement with a research advisory group (RAG) is one way for occupational therapists to incorporate meaningful consumer and community involvement in research.
- Authentic collaboration in a short‐term RAG was a novel experience for consumers, health service staff, and academics and resulted in many individual, partnership, and study benefits.
- Key success factors included careful RAG planning and recruitment, capable facilitation with support of a committed research team (inclusive of consumers), and empowering meeting processes and structures.

## 1 INTRODUCTION

Consumers, including patients, potential patients, carers, people who use health services, and community members, are willing and able to offer unique and valuable perspectives to research, beyond being consented study participants (Bird et al., 2020; de Iongh et al., 2021). Consumer and community involvement (CCI) is an Australian term that encompasses the range of consumer engagement activities across the research cycle (National Health and Medical Research Council & Consumers Health Forum of Australia, 2016). CCI has built momentum as a funding prerequisite for publicly sponsored research in Australia and internationally (Staniszewska et al., 2017). However, simply complying with policy often results in tokenistic efforts to ‘tick a box’ rather than addressing power dynamics and the achievement of authentic research partnerships (Atkin et al., 2020; Beeker et al., 2021; Happell et al., 2018). True partnerships involve bidirectional information flow and active consumer collaboration (Bird et al., 2020). Engagement of other research stakeholders such as clinicians, policy experts, and funding decision makers is also important (Young et al., 2021).

There have been calls to reduce the dominance of the professional voice in occupational therapy research by embracing CCI (Atkin et al., 2020; Gustafsson et al., 2019; Hammell et al., 2012). The *British Journal of Occupational Therapy* now requires a Patient and Public Involvement (equivalent term to CCI) statement for submissions (de Iongh et al., 2021). Joint ownership of occupational therapy research is key to achieving the espoused philosophies of person‐centred practice and consumer empowerment (Atkin et al., 2020; Hammell et al., 2012; Haywood et al., 2019). Moving occupational therapy research culture from conducting research *for* consumers, to *with* and *by consumers*, may reduce research waste (Cordier, 2021) and start to address the oppression people who experience health inequities encounter in everyday life and as research participants (Hammell et al., 2012; Magasi et al., 2021). However, a recent scoping review of health‐related research in the *Australian Occupational Therapy Journal* indicated that the profession has far to go in demonstrating meaningful CCI (Cox et al., 2020).

Research advisory groups (RAGs) are a common way to engage consumers, the community, and other stakeholders to influence research prioritisation and decision making (Isham et al., 2019; Kelly et al., 2017; Young et al., 2021). The role of a RAG is to ‘question and advise’ so that research is high quality and consumer focussed (Gordon et al., 2018). The empowering nature of collective discussion at RAGs, when time is taken to foster relationships and address power differentials, has been highlighted (Isham et al., 2019). However, the over‐representation of women and people from socio‐economically advantaged and ethnic majority groups in RAGs needs to be addressed (Islam et al., 2021; Musson et al., 2019). Examples of RAGs incorporating CCI in occupational therapy research include studies regarding university mental health curriculum (Arblaster et al., 2018), recreation and mental health services (Lauckner et al., 2018), and community age‐friendliness (Lauckner & Stadnyk, 2014).

Unfortunately, despite strong arguments for CCI in research, limited evidence exists to guide occupational therapists (Honey et al., 2019). In particular, there is a paucity of research regarding RAGs that are time or activity limited (Isham et al., 2019). Evaluation should investigate consumer training, support, and compensation provided, and impacts on learning, the researchers, and study outcomes (Pavarini et al., 2019). Additionally, group dynamics and power relationships in RAGs are under‐researched (Kelly et al., 2017). Thus, the aim of this study was to explore the processes and outcomes of a RAG partnership for an eDelphi study. The eDelphi study was international and refined a capability development framework for successful consumer and staff partnerships in health‐care quality improvement (QI). The methods, results, and implications of the eDelphi study are out of scope for this paper and have been reported elsewhere (Cox, Kendall, et al., 2022). Although the eDelphi study was interdisciplinary, the RAG was facilitated by an occupational therapist who was passionate about encouraging occupational therapy researchers to lead CCI. Thus, the research team, including two consumer co‐researchers, supported sharing findings and recommendations in an occupational therapy journal.

## 2 METHODS

### 2.1 Study design

Rapid ethnography is a research design, which originated from anthropology and uses field work over a compressed period of time, in this case, 6 months, to examine human experiences and practices by capturing social, cultural, and behavioural information (Vindrola‐Padros & Vindrola‐Padros, 2018). This design uses multiple data collection methods to increase understanding of how a cultural group (i.e., the RAG) interacts and attributes meaning to their collaboration and practices (Creswell & Poth, 2018). Examination of insiders' perspectives in ethnography has values, purpose, and power at its core (Monrouxe & Ajjawi, 2020), which aligned with the literature about CCI and was therefore suitable to address the research aim. Ethical approval was granted by the Metro South Health Human Research Ethics Committee (MS HREC/2019/QMS/52675) and the Griffith University Human Research Ethics Committee (GU Ref. No. 2019/659). Unfortunately, no consumers were remunerated for their engagement in this study due to a lack of funding. Two grant applications, which included a dedicated budget for consumer remuneration, were unsuccessful.

### 2.2 Researcher positionality

Positionality, or the researchers' influence on findings, includes preconceptions and philosophical stance and should be reported for transparency (Braun & Clarke, 2021). It is particularly relevant to ethnography where accounts are historically, politically, and personally situated (Monrouxe & Ajjawi, 2020). The research team was deeply committed to authentic consumer research partnerships and had already been collaborating for 2 years across several studies of the principal investigator's (PI) PhD. The PI was a metropolitan hospital and community occupational therapy manager in Australia with extensive CCI leadership experience. One consumer had 7 years' experience as a consumer advisor and consumer organisation representative at national, state, and local levels for health programmes, university research, and QI practice and policy. The second consumer had been the carer or family member of several patients in the local health service and a hospital volunteer. One PhD supervisor was an occupational therapy university academic and manager. The other supervisor had a psychology background and an academic appointment and was a senior researcher in a community rehabilitation service.

### 2.3 Participants and recruitment

RAG recruitment aimed to enhance the diversity of perspectives guiding the eDelphi study to ensure that the final capability framework had broad applicability and acceptability. Recruitment focussed on filling gaps in the research team lived experience, including First Nations peoples, cultural and linguistic diversity, medical and nursing professionals, non‐government consumer organisations, and mental health service providers and/or consumers. Eligibility criteria included at least 2 years' CCI experience in health‐care QI committee(s) and/or in QI projects, and/or authorship of a relevant peer reviewed publication(s), and/or recognised advisory role through a consumer‐led organisation supporting health service QI.

**FIGURE 1:**
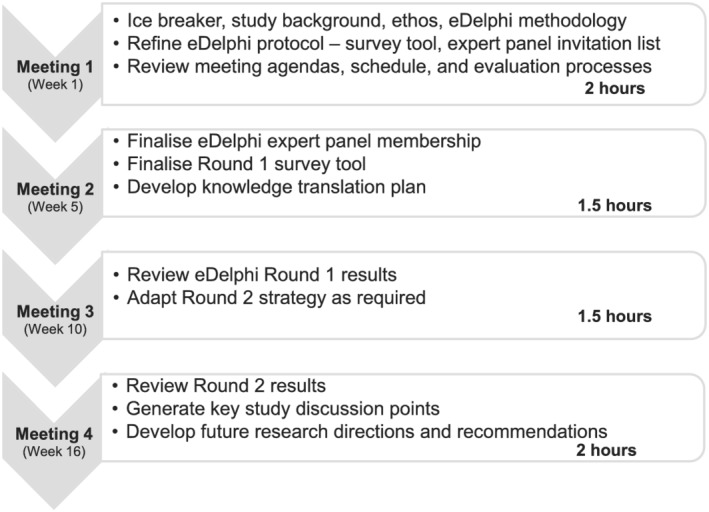
Research Advisory Group meeting schedule and topics

The research team aimed to recruit a RAG of 10–12 members (including the research team) to maximise diverse perspectives while accommodating the pragmatics of arranging meeting times and enabling meaningful, collaborative discussion. An invitation was emailed to 30 consumers and non‐consumers who were known to the research team or were part of their networks. It outlined the background and aims of the eDelphi study; RAG purpose and eligibility; and number, length, topic, and timing of proposed meetings (see Figure 1). A link to a 7‐minute video outlining the study was included. Interested people were invited to contact the PI to clarify the RAG purpose and expectations. An expression of interest (EOI) form requested demographic and eligibility screening information. It was also completed by the research team to reconfirm consent and enable inclusion of de‐identified details in findings.

### 2.4 Study process

There were four RAG meetings across 4 months as per Figure 1. Meeting 1 commenced with an icebreaker and established the eDelphi study background and ethos. The RAG then finalised the eDelphi panel invitation list and Round 1 survey tool, which had been co‐developed by the research team. Pre‐reading, learning, and reflective conversations focussed on eDelphi design (Woolcock, 2020), the scoping review that informed the eDelphi study (Cox, Molineux, et al., 2022), the International Association of Public Participation (IAP2) public participation spectrum (International Association for Public Participation Australasia, 2018), and best practice principles (Harrison et al., 2019) and ethical considerations for CCI in research (Leese et al., 2018). Meeting 2 developed a knowledge translation and dissemination plan and finalised the Round 1 survey tool and eDelphi panel membership. Meeting 3 sought advice regarding the Round 1 results interpretation and Round 2 planning. The fourth meeting considered Round 2 results interpretation, dissemination, implementation, and future research. Prioritised pre‐reading was emailed a week in advance and was estimated to take 1 to 2 hours. Table 1 outlines RAG meeting standing agenda items, which aimed to enhance genuine collaboration.

**TABLE 1:** Standing agenda items for the Research Advisory Group meetings

**Beginning of meeting**	Acknowledgement of country Check‐in—consent reconfirmation, training/learning, support—emotional and learning, confidentiality and anonymity, financial implications, reward and recognition, role boundaries and relationships, and best practice consumer engagement in research principles Review of agenda—meeting content and flow, and invitation to suggest changes Pre‐reading comments and reflections Decisions made and achievements as a result of, and since, previous RAG meeting
**End of meeting**	Actions required Keeping in touch Planning the next meeting Checkout—reflections on the meeting and ethical issues

Abbreviation: RAG, Research Advisory Group.

### 2.5 Data collection

Consistent with ethnography, multiple data collection methods enabled a rich, thick description of the RAG processes and outcomes through triangulation (Creswell & Poth, 2018; Monrouxe & Ajjawi, 2020). In‐depth focus groups are utilised in ethnography to empower collective negotiation of the meaning of shared experiences (Vindrola‐Padros & Vindrola‐Padros, 2018). However, consistent with the study philosophy, participants chose the final evaluation parameters, including a focus group or individual interviews, research team presence or absence, and facilitation by the PI or a PhD supervisor. They preferred a focus group (RAG‐FG) with the option of an individual interview (RAG‐I), and facilitation by a PhD supervisor, with the remaining research team absent to empower free discussion. RAG members were aware that the research team would access de‐identified written transcripts for analysis. The RAG focus group occurred 3.5 weeks after the last meeting. The research team participated in separate pre‐ and post‐RAG focus groups (RT‐FGpre and RT‐FGpost) 6 months apart and led by an external expert in qualitative participatory consumer‐focussed research.

The interview guide was the same for all focus groups, including what happened in the RAG or in planning, what did and did not work well, degree of RAG influence on the eDelphi study, benefits and challenges of involvement, ethical issues, and learning and support. Detailed written observations of interactions and direct quotes were documented in field notes by the PI for six research team meetings (RT‐FN1–6) and the four RAG meetings (RAG‐FN1–4). The research team also entered individual reflections into a monthly online log (RT‐Log). A reflexive diary was kept by the PI to increase self‐awareness of biases, values, and experiences (Creswell & Poth, 2018), which were discussed at research team meetings.

### 2.6 Data analysis

Qualitative analyses were iterative with four key papers informing reflexive discussions (Boivin et al., 2018; Harrison et al., 2019; International Association for Public Participation Australasia, 2018; Leese et al., 2018). Reflexive thematic analysis (Braun & Clarke, 2021) was chosen due to its previous successful use with the consumer co‐researchers (Cox et al., 2020). The following process was adopted: (1) data familiarisation and writing familiarisation notes; (2) systematic data coding; (3) generating initial themes from coded and collated data; (4) developing and reviewing themes; (5) refining, defining, and naming themes; and (6) writing the report. Additional strategies to enhance trustworthiness included data triangulation; a shared audit and memo trail; and documentation of key achievements, progress, and decisions for formal research meetings. Additionally, the PI and an academic researcher independently coded and then compared coding for the individual interview transcript and one field note. Minimal discrepancies were found and coding was updated, where required, following discussion. The PI independently coded the RAG focus group transcript. Then, according to their preference, the two consumer co‐researchers reviewed the coding with the PI at an online meeting with changes made following reflexive discussion. The PI then independently coded the remaining data and subsequently met online with the consumers to develop collated codes for approximately 50% of data. The PI then completed coding and generated preliminary themes. The whole team refined the themes, and the consumers led the theme naming discussions. The write‐up of themes was emailed to the RAG for member checking.

## 3 FINDINGS

Seven people from diverse backgrounds were recruited, which resulted in a RAG of 12 people, including the research team. Table 2 summarises RAG member characteristics. All recruitment aims were met except there was no doctor or person with mental health lived experience recruited. Three of the four health service staff were in specialist consumer partnership roles (25% of the RAG). Average age was 56.0 years (range 28–74 years), 75% (n = 9) were women, and all had a bachelor's degree or higher. RAG members had participated in CCI across the care continuum and regarding many health conditions. Over 90% had CCI experiences in committees and QI projects (91.7%, n = 11). Five (71.4%) of the seven non‐research team RAG members attended the focus group, and one participated in an individual interview. All research team members attended the separate pre‐ and post‐RAG focus groups. The GRIPP2 checklist is an evidence‐based, consensus‐informed guideline for reporting CCI in research (Staniszewska et al., 2017). The GRIPP2 short form for this study is included in Table 3. A summary of the extent of the consumer influence, and benefits and challenges of CCI for the eDelphi study are included in Supporting Information S1 to further demonstrate use of the GRIPP2.

**TABLE 2:** Characteristics of the Research Advisory Group

Characteristic	Research team (n = 5) (n)	Non‐research team (n = 7) (n)	Research advisory group (n = 12) % total RAG (n)
**Diversity indicators** ^a^			
Chronic condition	1	3	33.3% (4)
Older person (>65 years)	2	2	33.3% (4)
Living with a disability	1	1	16.7% (2)
Non‐English speaking	0	2	16.7% (2)
Carer of person with disability	1	0	8.3% (1)
Carer of older person	2	0	16.7% (2)
LGBTIQ+	1	0	8.3% (1)
Culturally diverse	0	3	25.0% (3)
Rural or remote	0	1	8.3% (1)
Australian Aboriginal	0	1	8.3% (1)
**Main partnership role for this RAG**			
Researcher or academic	2	1	25.0% (3)
Health‐care staff	1	3	33.3% (4)
Consumer	1	2	25.0% (3)
Consumer organisation representative	1	1	16.7% (2)
**Main health‐care context of partnership work**			
Hospital	1	1	16.7% (2)
Hospital and primary care	4	6	83.3% (10)
**Main clinical areas for partnership work** ^a^			
Generalist	1	5	50.0% (6)
Chronic disease	0	2	16.7% (2)
Rehabilitation or disability	2	0	16.7% (2)
Older persons	1	1	16.7% (2)
Cancer care	1	1	16.7% (2)
Mental health	0	1	8.3% (1)

Abbreviations: LGBTIQ+, lesbian, gay, bisexual, transgender, intersex, and queer +; RAG, Research Advisory Group.

^a^ Participants may have indicated more than one characteristic.

**TABLE 3:** GRIPP2 reporting checklist—Short form

Section and topic	Item	Section where reported and details
1. Aim	Reports the aim of consumer and community involvement^a^ (CCI) in the study	A clear RAG aim is included in the Methods: *RAG recruitment aimed to enhance the diversity of perspectives guiding the eDelphi study to ensure that the final capability framework had broad applicability and acceptability*. It is stated throughout the paper that two consumer co‐researchers were part of the research team and integral to study decision making from study inception with RAG recruitment focussing on filling gaps in research team lived experience.
2. Methods	Provides a clear description of the methods used for CCI in the study	The Methods include a clear description of the RAG recruitment and eligibility, meeting schedule and topics (see Figure 1), and standing agenda items at meetings (see Table 1). It also details how the consumer co‐researchers collaborated in all aspects of the RAG evaluation, including details of collaboration in data analysis.
3. Study results	Outcomes—reports the results of CCI in the study, including both positive and negative outcomes	The Findings incorporate detailed information regarding how the consumer co‐researchers and RAG had a high level of influence on decision making, data interpretation, and knowledge translation for the eDelphi study. Specific examples of how the consumer co‐researchers collaborated in RAG planning and supported the RAG facilitator are also included.
4. Discussion and conclusions	Outcomes—comments on the extent to which CCI influenced the study overall. Describes positive and negative effects	The influence of CCI is central to this paper. Supporting Information S1 summarises this information for the eDelphi study. The Discussion includes information about the time required but how this was offset by the considerable benefits. The benefits of challenging conversations resulting from diversity were also discussed.
5. Reflections/critical perspective	Comments critically on the study, reflecting on the things that went well and those that did not, so others can learn	Critical reflection is emphasised throughout the paper with sufficient details provided to promote learning.

Abbreviation: RAG, Research Advisory Group.

^a^ The GRIPP2 (Staniszewska et al., 2017) uses the term ‘patient and public involvement (PPI)’, which is a UK term equivalent to CCI.

Thematic analysis resulted in an overarching theme with four subthemes. Data sources are included in brackets. Five (71.4%) of the RAG members participated in member checking. They all provided strong support for the themes as an accurate summary of their experiences and outcomes.

### 3.1 Overarching theme: Different in so many ways

The overarching theme unites the subthemes and highlights that being part of a diverse RAG with an empowered group of consumers, academic researchers, and health service staff was a greatly valued and unique experience, which positively impacted on the participants and research outcomes. The high level of collaboration, emphasis on equal voices, and the iterative research process were praised by participants as ‘walking the talk of the [CCI] principles and practices’ (RAG‐FN4). ‘Everybody contributed … people felt comfortable … Some [other] groups get dominated, but it was a really equal group’ (RAG‐I). Treating the consumers as peers, rather than being ‘too deferential’, was commended as effective and unusual (RAG‐I). The research team was surprised that RAG members were so appreciative of the opportunity to contribute to a diverse and authentic research partnership, and a little disappointed that this experience was so unique, given they were all experienced in CCI. This finding reinforced to the research team that there is much work to be done to enhance CCI in research (RT‐FGpost).

The frequent limits on consumer empowerment at CCI meetings external to the study and limited consumer impact on outcomes were a common frustration voiced by participants. ‘Consumer engagement often stops at telling stories and then doesn't lead to design and evaluation. You reach a glass ceiling’ (RAG‐FN4). They were pleased that this was not their experience in the RAG where their input was genuinely deliberated, transparently decided on, and often actioned. Additionally, RAG members did not want to be ‘typecast in a box’ and often felt restricted to an assigned role in other committees (RAG‐FN2). This insight resulted in the eDelphi panel EOI form being changed so that more than one CCI role could be indicated. Another instance of RAG influence on the eDelphi study was the request for the eDelphi panel to rate QI partnership capabilities separately for consumers and health‐care staff. The research team experienced considerable philosophical discomfort with this suggestion, which was felt to perpetuate power imbalances. However, after lengthy discussion, it was actioned. The separate ratings led to interesting findings, which would not have been uncovered without an open, egalitarian attitude to RAG advice (RT‐FN2–6).

#### 3.1.1 Subtheme 1: Set up for success

A major contributing factor to the positive experiences of the RAG was that it was set up for success. Reflexive co‐planning included developing a clear RAG purpose, appropriate eligibility criteria, a welcoming and inclusive mindset, and a focus on diversity (RT‐FGpre). The high uptake of invitations may have resulted from the personalised but formal EOI based on existing relationships (RT‐Log). Additionally, local co‐presentations about prior studies of the PhD generated interest (RT‐FN2). Recruiting a combination of diverse, partnership‐ready consumers and non‐consumers was also invaluable as multiple perspectives were shared, discussed, and debated (RAG‐FG). Strategic recruitment meant that participant motivations were aligned with the study ethos and RAG purpose. ‘I want to practice my partnership skills, meet new people, and learn how the eDelphi process really works’ (RAG‐FN1, RT‐FGpost). The desire to ‘level the playing field’ (RAG‐FN4) in CCI in the local health service was frequently expressed as a motivator.

Collaborating as a research team to develop explicit and time limited meeting topics and expectations (see Figure 1) attracted participants: ‘… that was really detailed upfront—it would be four to five meetings potentially on these dates, this is what we're doing … that certainly made me want to be involved, knowing that … it would finish … you can pace yourself …’ (RAG‐I). RAG members appreciated being able to clarify requirements with the PI before committing (RAG‐FG). Pre‐reading, which included academic, health service, and consumer perspectives, also developed a ‘joint purpose’ and ‘… commonality in our thinking’ (RAG‐FG). RAG members understood that their role was to advise and to be solution focussed (RT‐FGpost).

#### 3.1.2 Subtheme 2: Authentic and capable facilitation

The facilitator's strong engagement skills were critical to enjoyment and successful outcomes. She expertly managed contributions by calling on others to ‘have their say’ if one person was beginning to dominate (RAG‐I). A consumer discussed that other committee chairs can sometimes alienate everyone because they do not have strong facilitation skills (RAG‐FN3). Confident but warm leadership of group direction and not being afraid to make a decision were also positive facilitator capabilities (RAG‐I). Being well organised, but adaptable within boundaries, nurtured a respectful, egalitarian environment (RAG‐FN3). These behaviours resulted from considerable facilitator self‐reflection and a purposeful approach to power‐sharing (RT‐FGpost). For example, the facilitator consciously managed her emotions and discussed options for resolution when a consumer questioned a fundamental aspect of the study (RAG‐FN2). The facilitator's experience as an occupational therapy clinician, manager, and leader in CCI contributed greatly to the RAG's success (RAG‐I, RT‐FGpost).

The capabilities of the whole research team, including the two consumers, were also vital. Reflexive discussion when co‐planning and then debriefing about RAG meetings was integral to a cycle of being responsive. The research team took a back seat to allow RAG members to speak as they were there to listen (RT‐FGpre and RT‐FGpost). Accommodating different communication and learning styles was also critical (RT‐FN1,6). Furthermore, the consumer co‐researchers modelled partnership behaviours by contributing thought‐provoking questions and insights, which promoted reflection and discussion (RT‐Log). The facilitator also relied on the academics to help refocus the RAG when conversations strayed out of scope (RT‐FGpost). The active engagement of the academics demonstrated that RAG advice was truly valued (RAG‐I).

#### 3.1.3 Subtheme 3: Structures and strategies for genuine partnerships

Specific meeting structures and processes employed to foster genuine engagement were refreshing and essential for establishing relationships, trust, and feeling respected (RAG‐FG). Participants felt that the ‘check‐in’ and ‘checkout’ about ethical issues on the standing agenda should feature in all meetings incorporating CCI (RAG‐FN1). Providing pre‐reading a week in advance and highlighting priority areas for discussion also promoted meaningful involvement (RAG‐FG). Celebrating achievements and specific RAG influences verbally and in meeting summaries was also beneficial. ‘When you saw that change made … that it is reflected … you feel like that discussion was really taken on board’ (RAG‐FG). Other effective strategies included being realistic about what could be achieved in meetings, maintaining communication between meetings with brief study updates, and sharing relevant external learning opportunities (RT‐Log, RT‐FN1,3,4).

A responsive, flexible, and individualised approach before, at, and in between meetings was also crucial. For example, three different preferences for receiving meeting documents were actioned (RAG‐FN1). Additionally, considerable time was taken to maximise collaboration for data interpretation by developing tables, written summaries, and graphs to meet individual needs (RT‐Log). Inclusiveness and responsiveness were encouraged by providing comprehensive preparation materials and individual meetings with the PI as required:

I couldn't attend the last [RAG meeting], so I wrote about four pages looking through all of the reading … and [the facilitator] got in touch with me afterwards. We had a separate meeting … it was included … which I think was very valuable, because, you know, you can write four pages and it kind of disappears somewhere, but certainly it didn't in this case. (RAG‐FG)

Participants talked about everyone ‘massaging the process’. ‘I think we could give ourselves a pat on the back that we were adaptable … [the facilitator] kept it within the bounds of the overall specifications but didn't try and keep it running on a set of absolutely unchangeable rails’ (RAG‐FG).

Experiential, shared learning with relevant short presentations and readings as the group progressed was favoured over a pre‐planned, once‐off training session (RAG‐FG). Conversely, one participant suggested that a preparatory workshop may be useful for people from First Nations or culturally diverse backgrounds (RAG‐FG). Videoconferencing was necessary due to COVID‐19, although most participants preferred face‐to‐face meetings (RAG‐FN1,4). The online experience was positive and reduced travel time, room booking problems, set‐up time, and parking issues (RAG‐FG, RT‐FGpost). However, videoconferencing prevented the research team from seeing if everyone was actively engaged (RT‐FN6).

#### 3.1.4 Subtheme 4: A ripple effect of benefits

The RAG collaboration had benefits for individuals, partnerships, and study outcomes both immediately and in the future. There was a common thread of enjoyment and satisfaction in learning. ‘I did feel a little bit of a personal accomplishment out of it, seeing the final product, and also for the opportunity to learn more about the Delphi study …’ (RAG‐FG). Another RAG member was inspired to use an eDelphi method in future research (RAG‐FN4). The RAG had many reflective conversations about consumer remuneration, equity, diversity, and the role of organisational leadership in CCI (RAG‐FN2–4). These rich discussions were beneficial to the research outcome and resulted in substantial co‐learning (RT‐Log). At times, deliberations were challenging with one consumer co‐researcher feeling ‘like an outsider’ during conversations about appropriate terminology regarding First Nations peoples and people who experience health inequities (RT‐FN6, RT‐FGpost). Experiencing authentic CCI built confidence and networks (RAG‐FG) and was anticipated to positively impact on future partnering.

I think it's given us, as consumers, a slightly different perspective … it's not just coming and telling the organisers of the group that this is good and that this isn't so good; there's a lot more to it than that … in a good partnership arrangement …. (RAG‐FG)

Influences on the eDelphi study have been described throughout the themes. The First Nations and culturally and linguistically diverse consumer perspectives were particularly valuable to the study and research team learning (RT‐FGpost). The RAG also brought a broader lens to knowledge dissemination and translation planning, including suggesting that a newsletter article summarising the eDelphi study findings be emailed to grass roots consumer groups (RAG‐FN2). All participants wanted the eDelphi study to make a difference and for findings to be ‘enacted and implemented’ (RAG‐FG), not ‘shelved as a nice piece of work’ (RAG‐FN4). Several RAG members talked about next steps and influencing their organisation or committees to implement the framework developed from the eDelphi study. Two participants offered to link the research team with other organisations for implementation (RAG‐FG, RAG‐FN4).

## 4 DISCUSSION

This study has demonstrated that with careful planning and recruitment, capable facilitation with the support of a committed research team (inclusive of consumers), and empowering meeting processes and structures, a short‐term RAG of 12 people resulted in many benefits to participants and enhanced research outcomes of an eDelphi study. These findings add to the limited research regarding RAGs and provide useful information for occupational therapists who have been called to demonstrate the profession's central tenets of person‐centeredness and power‐sharing through CCI in research (Cox et al., 2020; Hammell et al., 2012). The meaningful deliberations and interactions as peers between consumers, health‐care staff, and academic researchers were commended as a novel experience, despite all RAG members having been involved in other CCI work, albeit mainly in QI rather than research. These findings align with previous contentions that authentic CCI in research is not widespread and that RAGs are an effective strategy to foster mutually beneficial collaboration (Chan et al., 2021; Young et al., 2021). The combination of stakeholders was a strength. This supports previous findings that warn against separating clinicians and consumers in RAGs as it limits collaboration and can disempower and devalue consumer perspectives (Kelly et al., 2017). Collaborating with consumer co‐researchers for RAG planning, implementation, and evaluation from study inception was also central to success and is best practice (Bird et al., 2020; Harrison et al., 2019).

Participants highlighted the attractiveness of a short‐term RAG with a clear purpose, timelines, and expectations. This conflicts with the suggestion that single‐study RAGs can be transactional and limit relationship building (Samir et al., 2021) and opens the door for occupational therapists to consider short‐term RAGs as a valuable form of CCI. Formally recruiting RAG members and providing opportunities to clarify the study, roles, and expectations with the PI were also essential as noted previously (Harrison et al., 2019; Kelly et al., 2017). The value of having a clear RAG purpose also parallels literature that warns against ‘uncritical involvement’, which may waste resources, cause frustration, and even do harm (Isham et al., 2019; Musson et al., 2019). The diversity of RAG voices was celebrated as refreshing and valuable even though some conversations were challenging as found elsewhere (Isham et al., 2019; Islam et al., 2021). Occupational therapy researchers must continuously search for missing voices in CCI (Haywood et al., 2019). Interestingly, participants wanted their whole self recognised by not restricting them to one RAG role such as consumer or staff member. This influenced the eDelphi study and is mirrored in occupational therapy‐led research where acknowledging RAG members' complex identities minimised the ‘us–them’ gap between consumers and others and created a safer space for sharing personal experiences (Lauckner et al., 2018).

The critical role and capabilities of the occupational therapist RAG facilitator were emphasised, reinforcing that facilitators must be skilled in order to work in a truly collaborative, respectful, and inclusive way (Isham et al., 2019). Other critical facilitator capabilities included being confident, warm, organised, and flexible within the study boundaries. Additionally, occupational therapy researchers have highlighted that facilitation must be flexible, shifting from leadership to support, in order to empower the RAG and address changing priorities (Lauckner & Stadnyk, 2014). Constructive conflict during CCI can lead to the best research ideas and decisions (Staley et al., 2019), but researchers may have difficulty managing diverse and differing consumer perspectives and thus need facilitation training (Heckert et al., 2020). Occupational therapy researchers who are inexperienced in CCI may also benefit from joint mentorship by a consumer and a non‐consumer expert in order to learn from direct experience about the benefits of authentic research partnerships (Happell et al., 2018). A further key finding was the value of research team, RAG, and individual reflexivity and self‐reflection, which focussed on equalising power differentials by listening and promoting an open, transparent, and accepting attitude. This is reinforced by occupational therapy authors who emphasise the continuous and gradual process required to intentionally renegotiate power dynamics and build equal, productive, and transparent relationships with disability communities in research (Magasi et al., 2021). Occupational therapists value reflection in practice, but time limitations and low knowledge of theory are barriers (Knightbridge, 2019). Thus, it is recommended that occupational therapy researchers promote and implement reflexive practices in CCI and seek and/or provide learning and development for themselves and RAG members. All of the aforementioned capabilities have been recommended for successful QI partnerships (Cox, Molineux, et al., 2022) suggesting there are many commonalities between CCI for research and QI.

Standing agenda items, including ‘check‐ins’ and ‘checkouts’ about wellbeing, celebrating achievements, and explicitly highlighting RAG influences on the research, were useful, which concurs with other literature (Beeker et al., 2021; Kelly et al., 2017; Schilling et al., 2019). Taking time for informal conversations fostered a safe environment and mitigated the risks of emotional impacts on consumer partners (Beeker et al., 2021; Leese et al., 2018). Supporting flexible participation and adequate time for rich discussion were also essential (Musson et al., 2019). An icebreaker at the first meeting set the scene for a collegial and enjoyable atmosphere and is recommended. Pre‐reading and a short interactive presentation with reflective discussion about best practice principles for CCI in research (Harrison et al., 2019) initially and across meetings reinforced the RAG ethos and are advised for occupational therapy researchers. Providing short, targeted presentations with preparatory reading, along with the experiential learning resulting from collaboration, was effective for learning, although one participant advocated for a pre‐RAG workshop. In an interdisciplinary study, a formal workshop increased consumer self‐rated learning and confidence for research but did not meet all individual needs and may have inadvertently reinforced academic researcher power and knowledge (Richardson et al., 2019). Hence, emphasising experiential knowledge through mentoring, coaching, learning on the job, reflection cycles, and learning conversations may be more useful than formal training for research CCI (Staley et al., 2019). The right balance between formal training and other forms of learning to optimise authentic CCI in research requires further investigation.

RAG participation had immediate and ongoing benefits, including enjoyment, increased confidence to contribute to research, co‐learning, networking, and enhanced partnership capabilities as found by occupational therapy researchers (Haywood et al., 2019; Honey et al., 2019) and others (Gordon et al., 2018; Musson et al., 2019; Young et al., 2021). The many impacts of RAG deliberations on the eDelphi study were clear across the research cycle, including in the planning of knowledge dissemination and translation. This was a priority for RAG members to ensure that the eDelphi study results benefited consumers and were implemented in the real world. In order to make their research findings and recommendations accessible to consumers, occupational therapists must include CCI in knowledge translation as this is currently under‐reported (Cox et al., 2020). The RAG evaluation resulted in significant learning for both the research team and RAG members, which may positively influence future studies. In keeping with the recommendations above, a knowledge dissemination and translation plan for this RAG evaluation has been developed to ensure that specific strategies are used to share findings and recommendations with consumers. These benefits offset the increased time needed for the RAG, which concurs with other authors (Chan et al., 2021; Islam et al., 2021; Young et al., 2021). Although a resource‐intensive evaluation of all RAGs may not be realistic, at a minimum, critical group reflection ensures ongoing learning and improvement (Kelly et al., 2017; Pavarini et al., 2019) and may lead to enhanced consumer leadership as demonstrated in occupational therapy mental health research (Honey et al., 2019). An inclusive process evaluation can also build equal and reciprocal relationships leading to enhanced interactions and meeting attendance (Kelly et al., 2017; Schilling et al., 2019). Further research is needed to clarify optimal evaluation strategies for RAGs. The GRIPP2 (Staniszewska et al., 2017) guided reporting of CCI for this study and is recommended to occupational therapy researchers.

### 4.1 Limitations

RAGs and qualitative research are context specific, and thus, results may not be generalisable, particularly as all RAG members had substantial CCI experience. Engaging only RAG members who were highly educated and had access to videoconferencing may have also influenced findings. Additionally, no consumers on the RAG were paid, which may contribute to power imbalances (Bird et al., 2020). Remuneration was discussed extensively in the RAG with acknowledgement that it may enhance involvement of consumers who experience health inequities, but they also recognised the many other rewards from RAG participation. Additionally, if remunerating consumers was mandatory, this may preclude valuable partnership learning opportunities for research higher degree students who rarely have access to funding. Further advocacy is required to encourage universities and other organisations to recognise the importance of CCI in research and to fund consumer remuneration as mandatory irrespective of the career level of the researcher(s). Additionally, long‐term outcomes were anticipated rather than realised. Investigation of long‐term RAG and CCI outcomes, including impacts on future studies, relationships, policy, and research culture, is also needed.

## CONFLICTS OF INTEREST

There were no known conflicts of interest and no financial support for this study.

## AUTHOR CONTRIBUTIONS

R. C. led the study as part of her doctoral research, which was supervised by M. M. and M. K. All authors contributed to all aspects of the study across the research cycle. R. C. was responsible for the first draft of the manuscript with all authors revising and approving the final submission.

## Supporting information


Supporting Information S1

## Data Availability

The data that support the findings of this study are available on request from the corresponding author. The data are not publicly available due to privacy or ethical restrictions.

## References

[aot12830-bib-0001] Arblaster, K. , Mackenzie, L. , Matthews, L. , Willis, K. , Gill, K. , Hanlon, P. , & Laidler, R. (2018). Learning from consumers: An eDelphi study of Australian mental health consumers' priorities for recovery‐oriented curricula. Australian Occupational Therapy Journal, 65(6), 586–597. 10.1111/1440-1630.12518 30221773

[aot12830-bib-0002] Atkin, H. , Thomson, L. , & Wood, O. (2020). Co‐production in research: Co‐researcher perspectives on its value and challenges. British Journal of Occupational Therapy, 83(7), 415–417. 10.1177/0308022620929542

[aot12830-bib-0003] Beeker, T. , Glueck, R. K. , Ziegenhagen, J. , Goeppert, L. , Jaenchen, P. , Krispin, H. , Schwarz, J. , & von Peter, S. (2021). Designed to clash? Reflecting on the practical, personal, and structural challenges of collaborative research in psychiatry. Frontiers in Psychiatry, 12, 701312. 10.3389/fpsyt.2021.701312 34305686 PMC8292740

[aot12830-bib-0004] Bird, M. , Ouellette, C. , Whitmore, C. , Li, L. , Nair, K. , McGillion, M. H. , Yost, J. , Banfield, L. , Campbell, E. , & Carroll, S. L. (2020). Preparing for patient partnership: A scoping review of patient partner engagement and evaluation in research. Health Expectations, 23(3), 523–539. 10.1111/hex.13040 32157777 PMC7321722

[aot12830-bib-0005] Boivin, A. , Dumez, V. , Fancott, C. , & L'Espérance, A. (2018). Growing a healthy ecosystem for patient and citizen partnerships. Healthcare Quarterly, 21, 73–82. 10.12927/hcq.2018.25634 30566408

[aot12830-bib-0006] Braun, V. , & Clarke, V. (2021). One size fits all? What counts as quality practice in (reflexive) thematic analysis? Qualitative Research in Psychology, 18(3), 328–352. 10.1080/14780887.2020.1769238

[aot12830-bib-0007] Chan, M. , Scott, S. D. , Campbell, A. , Elliott, S. A. , Brooks, H. , & Hartling, L. (2021). Research‐ and health‐related youth advisory groups in Canada: An environmental scan with stakeholder interviews. Health Expectations, 24(5), 1763–1779. 10.1111/hex.13316 34288282 PMC8483214

[aot12830-bib-0008] Cordier, R. (2021). The research challenges we face: Identifying and minimising research waste. Australian Occupational Therapy Journal, 68(1), 1–2. 10.1111/1440-1630.12719 33415758

[aot12830-bib-0009] Cox, R. , Kendall, M. , Molineux, M. , Miller, E. , & Tanner, B. (2020). Consumer engagement in occupational therapy health‐related research: A scoping review of the *Australian Occupational Therapy Journal* and a call to action. Australian Occupational Therapy Journal, 68(2), 180–192. 10.1111/1440-1630.12704 33047341

[aot12830-bib-0010] Cox, R. , Kendall, M. , Molineux, M. , Miller, E. , & Tanner, B. (2022). Refining a capability development framework for building successful consumer and staff partnerships in healthcare quality improvement: A coproduced eDelphi study. Health Expectations, 1–17. 10.1111/hex.13499 PMC9327859 35472122

[aot12830-bib-0011] Cox, R. , Molineux, M. , Kendall, M. , Tanner, B. , & Miller, E. (2022). Co‐produced capability framework for successful patient and staff partnerships in healthcare quality improvement: Results of a scoping review. BMJ Quality and Safety, 31, 134–146. 10.1136/bmjqs-2020-012729 PMC8784995 34253682

[aot12830-bib-0012] Creswell, J. D. , & Poth, C. N. (2018). Qualitative inquiry and research design: Choosing among five approaches (4th ed.). Sage.

[aot12830-bib-0013] de Iongh, A. , Severwright, A. , & Taylor, J. (2021). Patient and public involvement statements in British Journal of Occupational Therapy: An important step. British Journal of Occupational Therapy, 84(8), 459–460. 10.1177/03080226211030103

[aot12830-bib-0014] Gordon, J. , Franklin, S. , & Eltringham, S. A. (2018). Service user reflections on the impact of involvement in research. Research Involvement and Engagement, 4, 11. 10.1186/s40900-018-0095-1 29600000 PMC5868049

[aot12830-bib-0015] Gustafsson, L. , Bourke‐Taylor, H. M. , & Pepin, G. (2019). Consumer and community co‐development in knowledge creation. Australian Occupational Therapy Journal, 66(4), 415–416. 10.1111/1440-1630.12605 31441074

[aot12830-bib-0016] Hammell, K. R. W. , Miller, W. C. , Forwell, S. J. , Forman, B. E. , & Jacobsen, B. A. (2012). Sharing the agenda: Pondering the politics and practices of occupational therapy research. Scandinavian Journal of Occupational Therapy, 19(3), 297–304. 10.3109/11038128.2011.574152 21631173

[aot12830-bib-0017] Happell, B. , Scholz, B. , Gordon, S. , Bocking, J. , Ellis, P. , Roper, C. , Liggins, J. , & Platania‐Phung, C. (2018). “I don't think we've quite got there yet”: The experience of allyship for mental health consumer researchers. Journal of Psychiatric and Mental Health Nursing, 25(8), 453–462. 10.1111/jpm.12476 29893451

[aot12830-bib-0018] Harrison, J. D. , Auerbach, A. D. , Anderson, W. , Weiss, R. , Fagan, M. , Hanson, C. , Carnie, M. , Wong, C. , Banta, J. , Symczak, G. , Robinson, E. , & Schnipper, J. (2019). Patient stakeholder engagement in research: A narrative review to describe foundational principles and best practice activities. Health Expectations, 22(3), 307–316. 10.1111/hex.12873 30761699 PMC6543160

[aot12830-bib-0019] Haywood, C. , Martinez, G. , Pyatak, E. A. , & Carandang, K. (2019). Engaging patient stakeholders in planning, implementing, and disseminating occupational therapy research. American Journal of Occupational Therapy, 73(1), 7301090010. 10.5014/ajot.2019.731001 30839255

[aot12830-bib-0020] Heckert, A. , Forsythe, L. P. , Carman, K. L. , Frank, L. , Hemphill, R. , Elstad, E. A. , Esmail, L. , & Lesch, J. K. (2020). Researchers, patients, and other stakeholders' perspectives on challenges to and strategies for engagement. Research Involvement and Engagement, 6, 60. 10.1186/s40900-020-00227-0 33042576 PMC7539495

[aot12830-bib-0021] Honey, A. , Berry, B. , Hancock, N. , Scanlan, J. , Schweizer, R. , & Waks, S. (2019). Using systematic collaborative reflection to enhance consumer‐led mental health research. British Journal of Occupational Therapy, 82(11), 666–674. 10.1177/0308022619862126

[aot12830-bib-0022] International Association for Public Participation Australasia . (2018). IAP2's Public Participation Spectrum. International Association for Public Participation Australasia. Retrieved October 24, 2020, from https://iap2.org.au/wp-content/uploads/2020/01/2018_IAP2_Spectrum.pdf

[aot12830-bib-0023] Isham, L. , Bradbury‐Jones, C. , & Hewison, A. (2019). Reflections on engaging with an advisory network in the context of a ‘sensitive’ research study. International Journal of Social Research Methodology, 22(1), 67–79. 10.1080/13645579.2018.1494971

[aot12830-bib-0024] Islam, S. , Joseph, O. , Chaudry, A. , Forde, D. , Keane, A. , Wilson, C. , Begum, N. , Parsons, S. , Grey, T. , Holmes, L. , & Starling, B. (2021). “We are not hard to reach, but we may find it hard to trust” …. Involving and engaging ‘seldom listened to’ community voices in clinical translational health research: A social innovation approach. Research Involvement and Engagement, 7(1), 1–15. 10.1186/s40900-021-00292-z 34174961 PMC8234650

[aot12830-bib-0025] Kelly, G. , Wang, S.‐Y. , Lucas, G. , Fraenkel, L. , & Gross, C. P. (2017). Facilitating meaningful engagement on community advisory committees in patient‐centered outcome research. Progress in Community Health Partnerships, 11(3), 243–251. 10.1353/cpr.2017.0029 29056616 PMC5679445

[aot12830-bib-0026] Knightbridge, L. (2019). Reflection‐in‐practice: A survey of Australian occupational therapists. Australian Occupational Therapy Journal, 66(3), 337–346. 10.1111/1440-1630.12559 30680734

[aot12830-bib-0027] Lauckner, H. , Litwiller, F. , Gallant, K. , White, C. , & Taylor, K. (2018). Deepening partnerships through co‐leadership: Integrating first voice perspectives into the Recreation for Mental Health (R4MH) Project. Scandinavian Journal of Occupational Therapy, 25(5), 325–334. 10.1080/11038128.2018.1502347 30280613

[aot12830-bib-0028] Lauckner, H. , & Stadnyk, R. (2014). Examining an occupational perspective in a rural Canadian age‐friendly consultation process. Australian Occupational Therapy Journal, 61(6), 376–383. 10.1111/1440-1630.12147 25308215

[aot12830-bib-0029] Leese, J. , Macdonald, G. , Kerr, S. , Gulka, L. , Hoens, A. M. , Lum, W. , Bao Chau, T. , Townsend, A. F. , & Li, L. C. (2018). ‘Adding another spinning plate to an already busy life’. Benefits and risks in patient partner–researcher relationships: A qualitative study of patient partners' experiences in a Canadian health research setting. BMJ Open, 8, e022154. 10.1136/bmjopen-2018-022154 PMC6104752 30121606

[aot12830-bib-0030] Magasi, S. , Angell, A. M. , Papadimitriou, C. , Ramirez, R. D. , Ferlin, A. , Reis, J. P. , & Wilson, T. (2021). Inside an occupational therapy–disability community partnership to promote health management: Ethnography of a research collaboration. American Journal of Occupational Therapy, 75(4), 7504180050. 10.5014/ajot.2021.045468 PMC8369666 34780614

[aot12830-bib-0031] Monrouxe, L. , & Ajjawi, R. (2020). Ethnography, methodology: Striving for clarity. Medical Education, 54(4), 284–286. 10.1111/medu.14129 32056236

[aot12830-bib-0032] Musson, L. S. , McDermott, C. J. , & Hobson, E. V. (2019). Exploring patient and public involvement in motor neuron disease research. Amyotrophic Lateral Sclerosis and Frontotemporal Degeneration, 20(7–8), 511–520. 10.1080/21678421.2019.1643373 31373235

[aot12830-bib-0033] National Health and Medical Research Council . & Consumers Health Forum of Australia . (2016). Statement on consumer and community involvement in health and medical research. https://www.nhmrc.gov.au/guidelines/publications/s01

[aot12830-bib-0034] Pavarini, G. , Lorimer, J. , Manzini, A. , Goundrey‐Smith, E. , & Singh, I. (2019). Co‐producing research with youth: The NeurOx young people's advisory group model. Health Expectations, 22(4), 743–751. 10.1111/hex.12911 31095837 PMC6737761

[aot12830-bib-0035] Richardson, C. , Akhtar, I. , Smith, C. , Edmondson, A. , Morris, A. , Hargreaves, J. , Rhodes, C. , & Taylor, J. (2019). Effective involvement: A report on the evaluation of a research awareness training package for public involvement in health research. Research Involvement and Engagement, 5, 21. 10.1186/s40900-019-0151-5 31223487 PMC6567900

[aot12830-bib-0036] Samir, N. , Diaz, A. M. , Hodgins, M. , Matic, S. , Bawden, S. , Khoury, J. , Eapen, V. , & Lingam, R. (2021). Speaking softly and listening hard: The process of involving young voices from a culturally and linguistically diverse school in child health research. International Journal of Environmental Research and Public Health, 18(11), 1–12. 10.3390/ijerph18115808 PMC8198054 34071425

[aot12830-bib-0037] Schilling, I. , Behrens, H. , Bleidorn, J. , Gágyor, I. , Hugenschmidt, C. , Jilani, H. , Schmiemann, G. , & Gerhardus, A. (2019). Patients' and researchers' experiences with a patient board for a clinical trial on urinary tract infections. Research Involvement and Engagement, 5(1), 38. 10.1186/s40900-019-0172-0 31798964 PMC6882213

[aot12830-bib-0038] Staley, K. , Cockcroft, E. , Shelly, A. , & Liabo, K. (2019). ‘What can I do that will most help researchers?’ A different approach to training the public at the start of their involvement in research. Research Involvement and Engagement, 5(1), 10. 10.1186/s40900-019-0144-4 30828464 PMC6383232

[aot12830-bib-0039] Staniszewska, S. , Brett, J. , Simera, I. , Seers, K. , Mockford, C. , Goodlad, S. , Altman, D. G. , Moher, D. , Barber, R. , Denegri, S. , Entwistle, A. , Littlejohns, P. , Morris, C. , Suleman, R. , Thomas, K. , & Tysall, C. (2017). GRIPP2 reporting checklists: Tools to improve reporting of patient and public involvement in research. Research Involvement and Engagement, 3(13), 11. 10.1186/s40900-017-0062-2 29062538 PMC5611595

[aot12830-bib-0040] Vindrola‐Padros, C. , & Vindrola‐Padros, B. (2018). Quick and dirty? A systematic review of the use of rapid ethnographies in healthcare organisation and delivery. BMJ Quality and Safety, 27(4), 321–330. 10.1136/bmjqs-2017-007226 29263139

[aot12830-bib-0041] Woolcock, K. (2020). Perspectives brief: The use of Delphi method for remote consultations. Australian Healthcare and Hospitals Association. Retrieved October 12, 2021, from https://ahha.asn.au/sites/default/files/docs/policy-issue/perspectives_brief_no_8._the_use_of_delphi_method_for_remote_consultations_1.pdf

[aot12830-bib-0042] Young, H. M. , Miyamoto, S. , Henderson, S. , Dharmar, M. , Hitchcock, M. , Fazio, S. , & Tang‐Feldman, Y. (2021). Meaningful engagement of patient advisors in research: Towards mutually beneficial relationships. Western Journal of Nursing Research, 43(10), 905–914. 10.1177/0193945920983332 33371791

