# Supplementary material for: Different in so many ways: Exploring consumer, health service staff, and academic partnerships in a research advisory group through rapid ethnography
Source: Aust Occup Ther J. 2022 Jul 24;69(6):676–88. doi: 10.1111/1440-1630.12830 (PMC10087328; doi:10.1111/1440-1630.12830)
Supplement: Supplementary file 1 — Supporting Information S1 [file AOT-69-676-s001.docx]

**Online Supporting Information Box 1: Summary of the extent of the consumer influence on the eDelphi study, and benefits and challenges of the consumer engagement**

| **Extent of consumer influence on the study**   - Two consumers were full members of the research team across the whole research cycle from development of the research proposal to manuscript writing. - RAG members had specific expertise regarding working with priority groups such as First Nations peoples; persons with a disability; and culturally and linguistically diverse individuals and communities. These perspectives enhanced interpretation of study findings. - All consumers emphasised that organisational culture and leadership are fundamental to enabling effective partnerships and this perception was central to understanding the results. - All consumers provided unique insights into the most effective strategies for knowledge dissemination and translation with an added emphasis on consumer confidence building. |
| --- |
| **Benefits of the consumer research collaboration**   - Members of the research team and RAG reported gaining additional capabilities regarding co-design and co-production which could be used to enhance future partnerships. - The consumer co-researchers were fundamental to all study decision making. This aligned with the philosophical paradigm of the topic and enhanced study credibility. - Adding consumers from additional diverse backgrounds through the RAG ensured that a greater range of partnership perspectives and expertise were incorporated. - Following suggestions from the RAG, additional contacts were added to the eDelphi panel invitation list and the expression of interest form was changed to enable choice of more than one role in CCI. - The RAG recommended that the international panel rate the importance of the capability domains and descriptions separately for consumers and healthcare staff. This led to important findings which reflected the realities of partnership experiences. - The role of grass roots consumer organisations in spreading the reach of research and QI was emphasised by the consumers. |
| **Challenges of the consumer research collaboration**   - Adding a RAG lengthened the timeframes of the eDelphi study and was time consuming in terms of recruitment, preparation, facilitation, and data collection. - Ensuring that the communication preferences of a larger group of consumers were met required flexibility and considerable additional time. - The local research governance office required the consumer co-researchers to sign a research agreement which was not entirely suitable. This resulted in advocacy and development of an improved agreement for the future. - The international focus of the eDelphi study was challenging for some consumers on the RAG as their primary motivation was on achieving local improvements and change. - The lack of funding for consumer compensation was acknowledged as potentially limiting engagement of consumers from diverse socioeconomic backgrounds, however, reimbursement of expenses was available. - The requirement for all meetings to be via videoconference due to COVID-19 restrictions may have limited the diversity of consumers involved. |
